# Study on the correlation between alkaloids and tastes of *Coptis* Rhizome from four species based on UHPLC-QQQ-MS/MS combined with electronic tongue technique

**DOI:** 10.3389/fpls.2024.1496789

**Published:** 2024-11-07

**Authors:** Yufeng Huang, Wenhui Luo, Wenhan Pei, Dongmei Sun, Hua Zhou, Fan He

**Affiliations:** ^1^ Guangdong Provincial Hospital of Chinese Medicine, The Second Affiliated Hospital of Guangzhou University of Chinese Medicine, Guangdong, China; ^2^ School of Chinese Materia Medica, Guangdong Pharmaceutical University, Guangdong, China; ^3^ Technique Center, Guangdong Yifang Pharmaceutical Co., Ltd, Foshan, China; ^4^ The Ministry of Education (MOE) Key Laboratory of Standardization of Chinese Medicines and the State Administration of Traditional Chinese Medicine (SATCM) Key Laboratory of New Resources and Quality Evaluation of Chinese Medicines, Institute of Chinese Materia Medica, Shanghai University of Traditional Chinese Medicine, Shanghai, China

**Keywords:** *Coptis* rhizome, electronic tongue, bitterness, UHPLC-QqQ-MS/MS, berberine, quality evaluation

## Abstract

**Objective:**

Taste is one of the vital indicators for the quality evaluation of *Coptis* rhizome (CR), but the traditional taste evaluation lacks objectivity. By establishing the correlation between CR’s tastes and alkaloids, an objective basis for the taste evaluation was established.

**Methods:**

Ultra-high performance liquid chromatography tandem triple quadrupole mass spectrometry (UHPLC-QQQ-MS/MS) and electronic tongue technique were performed to determine ten alkaloid contents and eight tastes from *Coptis chinensis* rhizome, *Coptis deltoidea* rhizome, *Coptis teeta* rhizome, and *Coptis japonica* rhizome, respectively. Combined with multivariate statistical analysis, we established models to discriminate the alkaloid contents and tastes of CR, screened the differential alkaloids and tastes, and performed Pearson’s correlation analysis on the results of alkaloids and tastes.

**Results:**

1) According to the previous UHPLC-QQQ-MS/MS method established by our research group, the contents of ten alkaloids of the four species of CR were quantified, of which jatrorrhizine, columbamine, and magnoflorine were the differential alkaloids of the four species. 2) The electronic tongue technique realized the objectification of CR’s tastes and distinguished the species of CR based on the tastes of aftertaste-A, sourness, bitterness, and richness. 3) Pearson’s correlation analysis shows the bitterness of CR was mainly manifested as aftertaste-B, indicating the higher the aftertaste-B value, the higher the berberrubine content. Astringency and aftertaste-A could be suggested as quality evaluation indexes of CR, due to the positively correlated or significantly positively correlated with coptisine, epiberberine, berberine, and palmatine, respectively.

**Conclusion:**

Electronic tongue technique has successfully achieved the objectification of the tastes of CR, and combined with UHPLC-QQQ-MS/MS technique for alkaloid quantification and correlation research, it provides a new idea for the quality evaluation of CR.

## 1 Introduction

*Coptis* rhizome (CR) is a multi-source herbal medicine that has significant clinical use. CR is derived from the species of *Coptis chinensis* Franch. (CC), *Coptis deltoidea* C.Y. Cheng *et* Hsiao (CD), and *Coptis teeta* Wall. (CT). The rhizome of *Coptis japonica* Makino. (CJ) is also used as CR in the Japanese Pharmacopoeia, the Korean Pharmacopoeia, and the international standard ISO 7177:2023.

The traditional view considers the tastes of CR to be highly correlated with the quality of the herb, and “bitter taste” has been taken as the basis for the superior quality of CR. The taste of CR has also been described in several pharmacopeias, but the traditional identification of CR relies on personal experience and lacks objective quantitative indicators (Xu et al., 2016). The electronic tongue, also known as bionic taste, is an artificial taste system designed to simulate the mechanism of human taste perception. It can realize food and drug samples’ objective and quantitative taste (Wasilewski et al., 2019; Lu et al., 2022). The technology has begun to be applied in Chinese medicines, realizing the digitization and objectification of sensory evaluation of Chinese herbal medicines (Li et al., 2016; Yaroshenko et al., 2016). Liang et al. (2014) measured the bitter degree of six alkaloids isolated from *Coptis chinensis* rhizome using the electronic tongue technique. Still, there is a lack of objectification of CR’s other taste sensations. Therefore, the SA-402B taste analysis system used in this study can simultaneously objectify eight tastes of samples, which make up for the shortcomings of traditional taste identification methods for CR.

Alkaloids are CR’s primary pharmacological active ingredients, with effects of antibacterial action, anti-inflammation, antioxidant, antitumor, immune regulation, hypolipidemic, and hypoglycemic (Wang et al., 2019). In modern studies, quality evaluation of CR is focused chiefly on chemical compound analysis. Berberine, epiberberine, coptisine, and palmatine, as representatives of the pharmacodynamic active ingredients of CR, have been used as recognized indicators for the quality evaluation of CR (China Pharmacopoeia Commission, 2020). However, chemical analysis techniques such as high performance liquid chromatography (HPLC) and ultra-high performance liquid chromatography tandem mass spectrometry (UHPLC-MS) are complicated to operate and have high detection costs, leading to limitations in practical application (Wang P. et al., 2024). In the trend of paying more attention to the overall quality evaluation, there is a lack of correlation studies between the traditional subjective evaluation of CR (tastes) and its objective evaluation (alkaloids).

Therefore, in this study, we objectively evaluated the quantitative characterization of CR’s “taste” by the electronic tongue technique and established a digital evaluation method for the specificity of CR’s “taste”, which can succeed in species identification of CR. Then, a “taste-alkaloid” correlation analysis was carried out by combining the results of ten alkaloid contents in CR by ultra-high performance liquid chromatography tandem triple quadrupole mass spectrometry (UHPLC-QQQ-MS/MS), which provides a new idea for the quality evaluation of CR.

## 2 Materials and methods

### 2.1 Materials and reagents

A total of 18 batches of CR were obtained from four plant sources, and the specific information is shown in **Table 1**. Professor Xian-you Qu from Chongqing Academy of Chinese Materia Medica and senior agronomist Wei Dai from Mianyang Academy of Agricultural Sciences identified all CR samples. Chemical reference substances (CRS), such as magnoflorine, tetrahydricheilanthifolinium, coptisine, epiberberine, columbamine, jatrorrhizine, berberrubine, berberine, palmatine, berlambine (purity ≥98%, HPLC grade), were purchased from Shanghai Yuanye Bio-Technology Co., Ltd. (Shanghai, China). The HPLC-graded methanol, acetonitrile, formic acid, and other chemical reagents were obtained from Fisher Scientific (Pittsburgh, PA, USA). The water was obtained from a Milli-Q water purification system (Millipore, Bedford, MA, USA).

**Table 1:** Sample information of *Coptis* rhizome.

Sample ID	Plant source	Place of origin
CC-1	*Coptis chinensis* Franch.	Pengzhou, Sichuan, China
CC-2	*Coptis chinensis* Franch.	Jiannan, Hubei, China
CC-3	*Coptis chinensis* Franch.	Shizhu, Chongqing, China
CC-4	*Coptis chinensis* Franch.	Emei, Sichuan, China
CC-5	*Coptis chinensis* Franch.	Shizhu, Chongqing, China
CC-6	*Coptis chinensis* Franch.	Shizhu, Chongqing, China
CC-7	*Coptis chinensis* Franch.	Shizhu, Chongqing, China
CC-8	*Coptis chinensis* Franch.	Shizhu, Chongqing, China
CC-9	*Coptis chinensis* Franch.	Shizhu, Chongqing, China
CD-1	*Coptis deltoidea* C.Y.Cheng et Hsiao.	Meishan, Sichuan, China
CD-2	*Coptis deltoidea* C.Y.Cheng et Hsiao.	Meishan, Sichuan, China
CD-3	*Coptis deltoidea* C.Y.Cheng et Hsiao.	Meishan, Sichuan, China
CT-1	*Coptis teeta* Wall.	Gaoli, Yunnan, China
CT-2	*Coptis teeta* Wall.	Nujiang, Yunnan, China
CT-3	*Coptis teeta* Wall.	Dehong, Yunnan, China
CJ-1	*Coptis japonica* Makino.	Tokyo, Japan
CJ-2	*Coptis japonica* Makino.	Kyoto, Japan
CJ-3	*Coptis japonica* Makino.	Kyoto, Japan

### 2.2 Preparation of reference solutions and test sample solutions

The CRS solutions were accurately weighed and dissolved in methanol. Their concentrations for working solution were as follows: magnoflorine (6.34 μg/mL), tetrahydricheilanthifolinium (5.38 μg/mL), coptisine (36.5 μg/mL), epiberberine (34.7 μg/mL), columbamine (5.82 μg/mL), jatrorrhizine (12.35 μg/mL), berberrubine (4.05 μg/ml), berberine (32.3 μg/mL), palmatine (27.3 μg/mL), berlambine (2.86 μg/mL).

Accurately weighed 0.2 g CR powder and placed it in a stoppered conical flask, accurately added 50 mL of methanol-hydrochloric acid (100: 1) solution to the flask followed by ultrasonic treatment for 30 min. Made up for the mass loss with methanol and centrifuged it. Took 2 mL of filtrate and put it into a 10 mL volumetric flask and set volume with methanol; Then, filtered through a 0.22 μm micropore membrane. The filtrate was used for liquid chromatography tandem mass spectrometry (LC-MS) analysis.

The samples used for the electronic tongue test were prepared as follows: weighed accurately 3.0 g of CR powder and placed in a conical flask, added 50 mL of ultrapure water; Extracted by ultrasonic for 30 min, then centrifuged at 4000 rpm for 5 min. The supernatant was taken for electronic tongue analysis. Potassium chloride and tartaric acid were taken as the reference solution for the electronic tongue. The response values of each sensor of the reference solution were taken as the base point. Since the reference solution contained acid and salt components, the base points for sourness and saltiness were -13 and -6, and the base points for the rest of the tastes were zero. All solutions were stored in a refrigerator at 4°C until analysis.

### 2.3 Apparatus and conditions of LC-MS

LC-MS analysis was performed on an Agilent 1290 series ultra-high performance liquid chromatography system tandem 6460 triple quadrupole mass spectrometer (Agilent Technologies, Santa Clara, CA, USA), which was previously reported by our team (He et al., 2022). In brief, the chromatographic separation was carried out at 30°C on a Waters Acquity UPLC CSH C_18_ column (2.1×100 mm, 1.7 μm, Waters, Milford, MA, USA). A mobile phase consisting of 0.1% acetic acid (A) and acetonitrile (B) in a gradient elution manner as follows: 10-14% B at 0-3 min, 14-16% B at 3-9 min, 16-25% B at 9-13 min, 25-80% B at 13-14 min, 80% B at 14-16 min. The sample injection volume was 2 μL, and the flow rate was 0.35 mL/min. Mass detection was performed on an electrospray ionization (ESI) source. The mass spectrum was chosen in positive mode, and the mass spectra were acquired in multiple reaction monitoring (MRM) mode. The drying gas (N_2_) flow rate was 11.0 L/min; the drying gas temperature was 300°C; the nebulizer was 15 psig, and the capillary voltage was 4000 V. The parent ion, daughter ion, fragment voltage, and collision energy were adjusted to obtain the highest abundance of each analyte. The method validation was investigated according to the Chinese Pharmacopoeia (China Pharmacopoeia Commission, 2020), and the results met the requirement of sample chemical analysis.

### 2.4 Apparatus and conditions of electronic tongue

SA-402B taste analysis system (Intelligent Sensor Technology, Inc., Japan) was adopted to evaluate the taste characteristics of CR in this study. It contained five taste sensors (**Table 2**) and two reference electrodes (Ag/AgCl).

**Table 2:** Array performance of electronic tongue sensor.

Sensor name	Initial taste	After taste
CA0	sourness	–
C00	bitterness	aftertaste-B
AE1	astringency	aftertaste-A
AAE	umami	richness
CT0	saltiness	–

The electronic tongue was activated, calibrated, and equilibrated to the available conditions; then samples were measured according to the recently reported method (Wang JW. et al., 2024). Briefly, the electronic tongue sensor determined the membrane potential of each sample, collecting one data per second for 30 s with a stirring rate of 1 r/s. It was washed in the reference solution for 3 s and inserted into a new reference solution to test the retort for 30 s. Each sample was measured in parallel four times and analyzed.

The precision of the instrument was investigated. The test solution of sample CC-1 was taken, and the sample was repeated five times using the electronic tongue to measure the response value of each sensor and calculate the relative standard deviation (RSD). The RSD of each sensor response value was less than 2.0%, indicating good precision of the instrument.

### 2.5 Statistical analysis

The results of tastes and alkaloid contents of CR were subjected to orthogonal partial least squares discriminant analysis (OPLS-DA) as well as cluster analysis using SIMCA 18.0 software (Umetrics AB, Umea, Sweden). Pearson’s correlation analysis between the taste values and alkaloid contents of CR was performed by the Scientific Platform Serving for Statistics Professional (SPSSPRO) platform (Scientific Platform Serving for Statistics Professional, 2021).

## 3 Results

### 3.1 LC-MS analysis of CR

**Figure 1:**
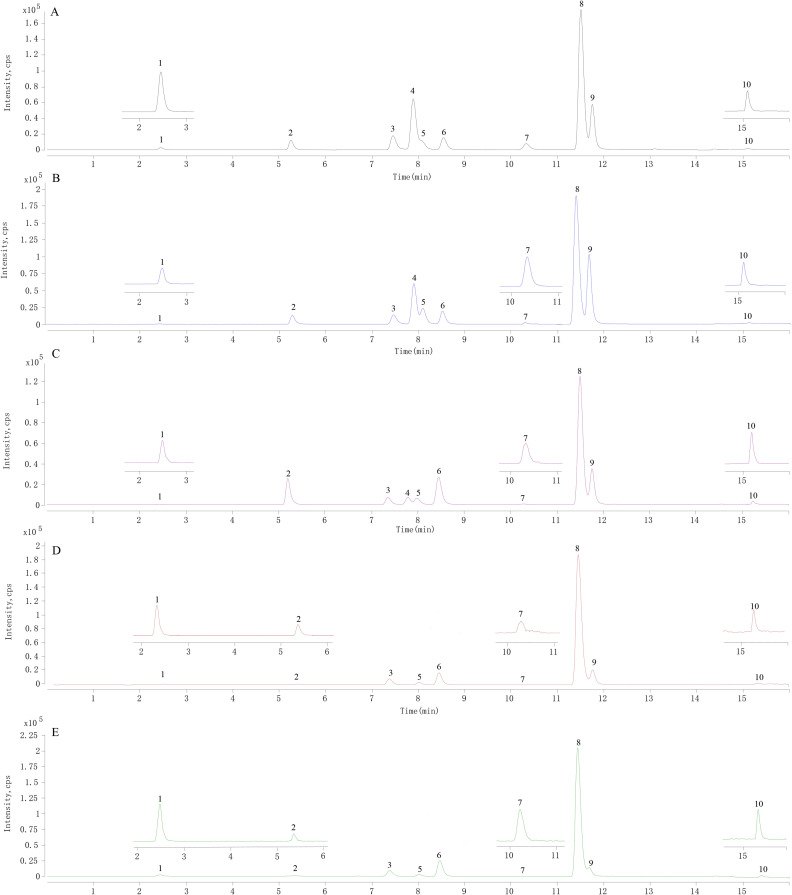
The representative MRM chromatograms of mixed reference solution and samples of *Coptis* rhizome. **(A)** mixed reference solution, **(B)** test solution of *Coptis chinensis* rhizome, **(C)** test solution of *Coptis deltoidea* rhizome, **(D)** test solution of *Coptis teeta* rhizome, and **(E)** test solution of *Coptis japonica* rhizome. 1. magnoflorine, 2. tetrahydricheilanthifolinium, 3. coptisine, 4. epiberberine, 5. columbamine, 6. jatrorrhizine, 7. berberrubine, 8. berberine, 9. palmatine, 10. berlambine.

The UHPLC-QQQ-MS/MS method successfully determined the contents of ten alkaloids in eighteen batches of CRs from four species. The chromatograms of the samples are shown in (**Figure 1**), and the content results are shown in **Table 3**. Epiberberine was not found in CRs derived from CT and CJ. Berberine is the alkaloid with the highest average content (7.95 ± 2.42%) in all CR samples.

**Table 3:** The contents of ten alkaloids from four species of *Coptis* rhizome.

Sample ID	Magnoflorine (%)	Tetrahydricheilanthifolinium(%)	Coptisine (%)	Epiberberine(%)	Columbamine(%)	Jatrorrhizine(%)	Berberrubine(%)	Berberine (%)	Palmatine(%)	Berlambine(%)
CC-1	0.821	2.89	2.76	1.61	0.912	0.600	0.211	9.44	2.46	0.0558
CC-2	1.01	3.77	3.66	1.52	0.938	0.673	0.285	11.1	2.91	0.0449
CC-3	0.728	4.01	3.16	1.86	0.984	0.547	0.547	10.2	2.74	0.0533
CC-4	0.927	3.54	3.28	1.55	0.960	0.645	0.645	10.2	2.87	0.123
CC-5	0.602	2.52	2.38	1.44	0.673	0.473	0.473	7.51	2.03	0.0246
CC-6	0.803	3.15	3.19	1.93	0.980	0.775	0.775	9.93	3.13	0.0900
CC-7	0.964	3.58	4.02	1.73	1.13	0.804	0.804	11.5	3.02	0.0995
CC-8	0.737	3.38	3.27	2.02	0.911	0.638	0.253	10.0	2.69	0.0293
CC-9	0.817	3.31	3.30	1.70	0.960	0.610	0.550	9.98	2.75	0.0630
CD-1	0.161	0.466	1.18	0.115	0.186	0.723	0.0136	3.85	0.504	0.0654
CD-2	0.157	0.451	1.20	0.119	0.150	0.645	0.00822	3.37	0.382	0.0872
CD-3	0.248	0.672	1.65	0.207	0.163	0.787	0.0129	4.45	0.469	0.0462
CT-1	0.266	0.0105	1.18	0	0.567	0.472	0.00305	6.80	0.318	0.0144
CT-2	0.374	0.0112	1.30	0	0.739	0.616	0.0123	6.91	0.390	0.0603
CT-3	0.359	0.0111	1.09	0	0.630	0.525	0.00445	7.26	0.381	0.137
CJ-1	0.411	0.00908	0.977	0	0.0637	0.612	0.0646	6.68	0.0720	0.0372
CJ-2	0.969	0.00577	1.77	0	0.105	0.802	0.0112	7.85	0.179	0.0451
CJ-3	0.754	0.00189	0.226	0	0.106	0.916	0.0156	6.02	0.178	0.0294
Minimum	0.157	0.541	0.226	0	0.0637	0.472	0.00100	3.37	0.0720	0.0144
Maximum	1.01	3.77	4.02	2.02	1.13	0.916	0.804	11.5	3.13	0.137

### 3.2 Taste analysis of CR

**Figure 2:**
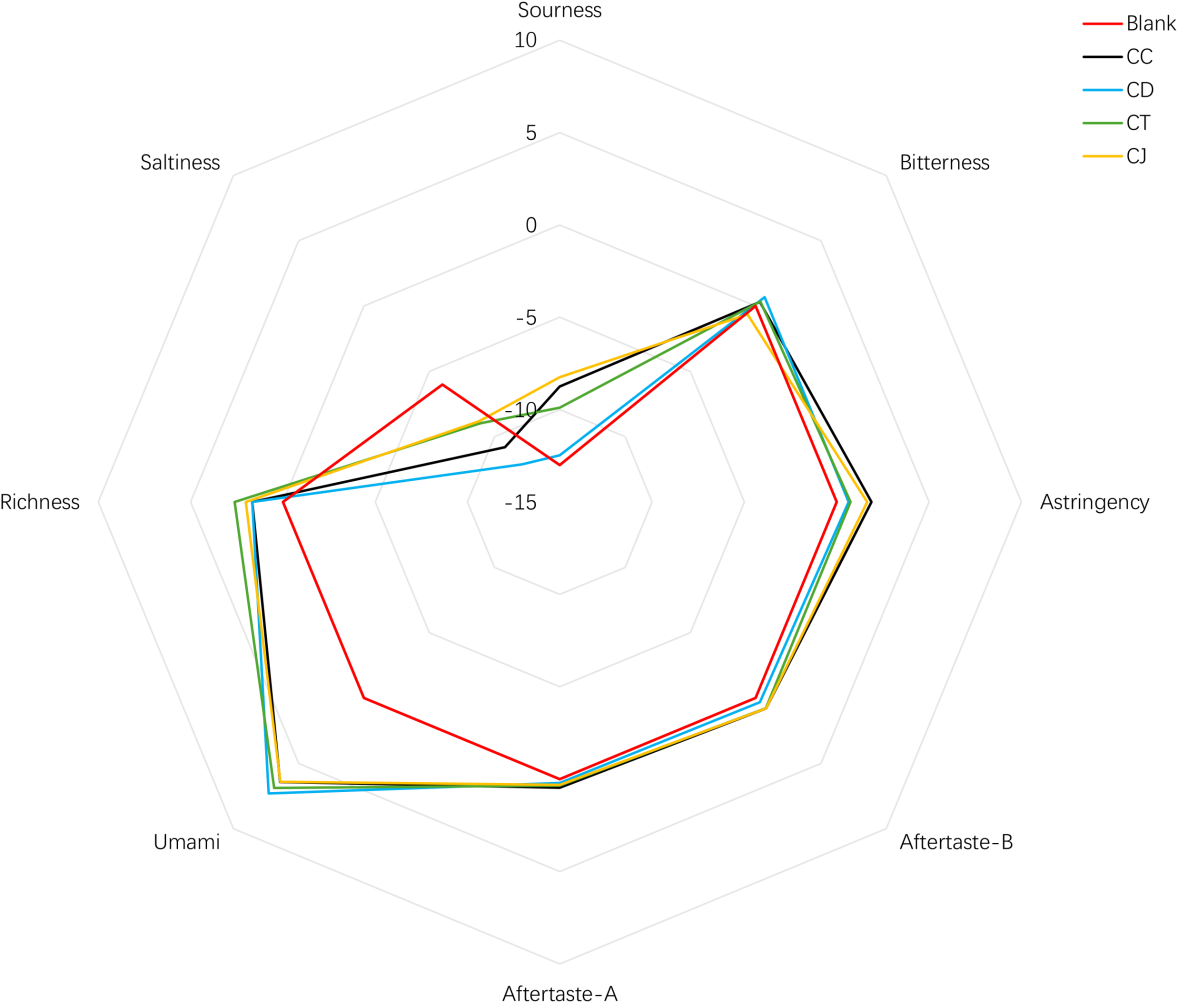
The radar chart of *Coptis* rhizome’s tastes.

The five sensors of the SA-402B taste analysis system successfully detected eight tastes in 18 batches of CR samples. The taste indices of CR are achieved through quantification and visualization (**Figure 2**). There were differences in the different taste values for CR. Umami, sourness, and richness were the tastes with CR’s top three response values. The mean value of saltiness for all samples, as well as that of bitterness for CJ, was below the no-taste point of the sensor response value. Unfortunately, the radar chart (**Figure 2**) cannot distinguish well among the different species of CR, which needs to be further analyzed by statistical methods.

### 3.3 OPLS-DA for CR

**Figure 3:**
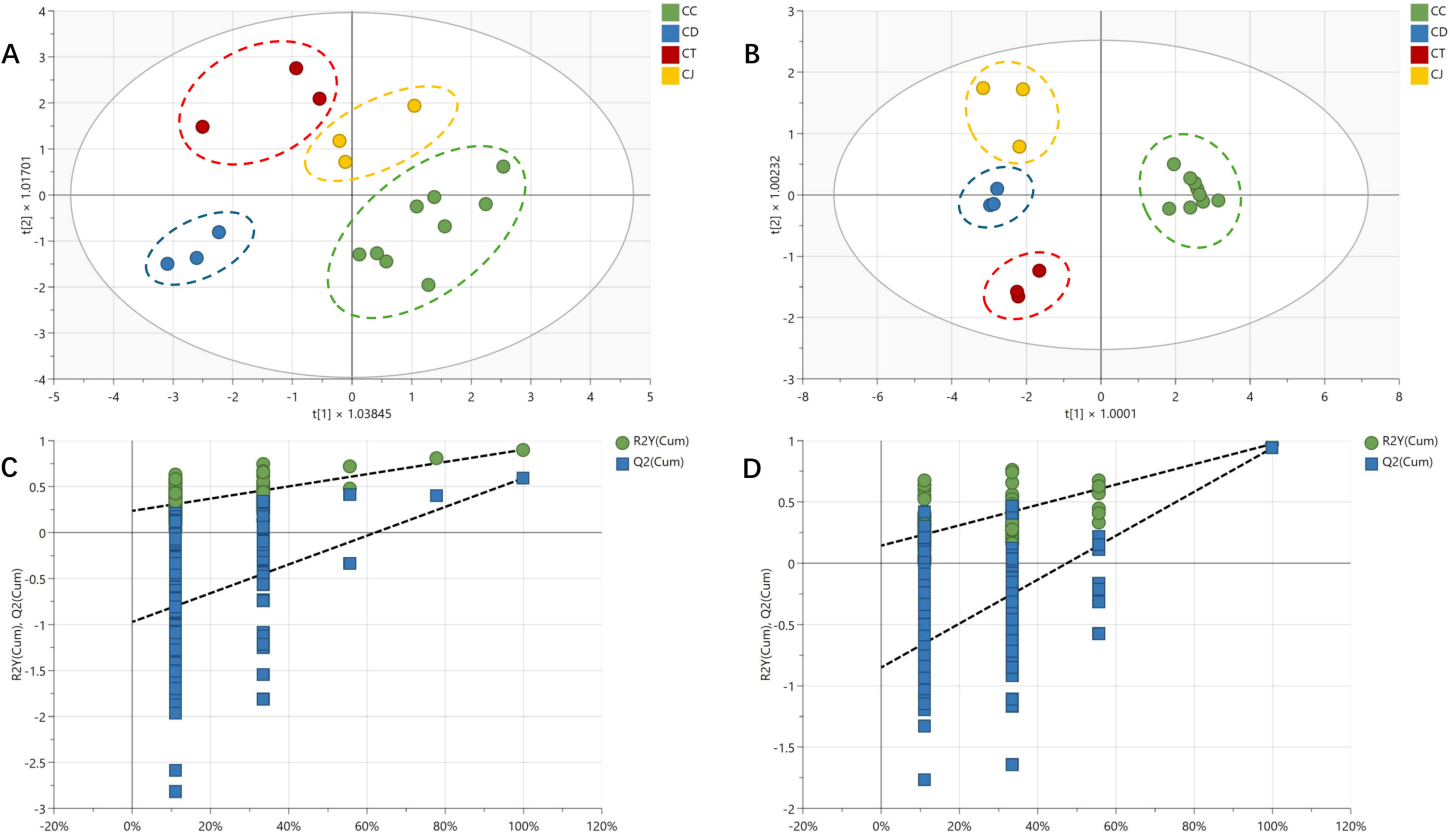
The OPLS-DA model diagram of tastes and alkaloid contents for *Coptis* rhizome. **(A)** core scatter plot of tastes; **(B)** core scatter plot of alkaloid contents; **(C)** correlation plot between original Y-vector and permuted Y-vector of tastes; **(D)** correlation plot between original Y-vector and permuted Y-vector of alkaloid contents.

The OPLS-DA of tastes and alkaloid contents for CR were well performed by SIMCA 18.0 software, respectively. **Figures 3A, C** show that the taste results determined by the electronic tongue can successfully differentiate four species of CR (R2X 0.928, R2Y 0.78, Q2 0.395). Aftertaste-astringency (aftertaste-A), sourness, bitterness, and richness played the key roles (VIP >1). As well as, **Figures 3B, D** show that the alkaloid contents of CR determined by UHPLC-QQQ-MS/MS can also be used to differentiate CR from the four species (R2X 0.928, R2Y 0.671, Q2 0.57), with jatrorrhizine, columbamine, and magnoflorine playing the key roles (VIP >1).

### 3.4 Hierarchical cluster analysis for CR

**Figure 4:**
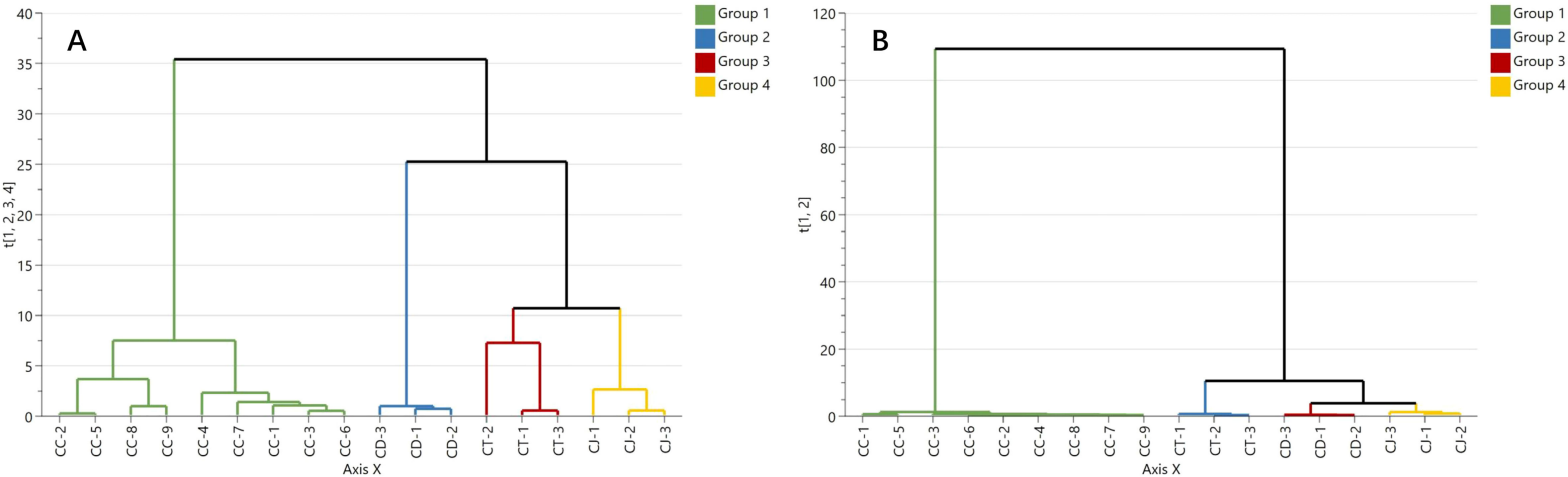
The dendrogram of hierarchical cluster analysis according to the tastes **(A)** and alkaloid contents **(B)** for CR.

A hierarchical cluster analysis of tastes and alkaloid contents for CR was performed by SIMCA 18.0 software, respectively. HCA result shows that the taste results (**Figure 4A**) and alkaloid contents (**Figure 4B**) successfully distinguished the four groups, respectively. The grouping results were consistent with those of CR species, which was the same as the results of Clause 3.3.

### 3.5 Correlation analysis of tastes and alkaloids for CR

Pearson’s correlation analysis of tastes and alkaloid contents for CR was carried out using the SPSSRO platform, and a significant correlation was observed in the results in **Table 4**. Uncommonly, the bitterness of CR was negatively correlated with jatrorrhizine (*P <*0.05), while aftertaste-bitterness (aftertaste-B) was positively correlated with berberrubine (*P <*0.05). The results suggested that the bitterness of CR was mainly manifested as aftertaste-B, indicating the higher the aftertaste-B value, the higher the berberrubine content. In addition, astringency was positively correlated with coptisine and palmatine (*P <*0.05) and significantly positively correlated with epiberberine and berberine (*P <*0.01); meanwhile, aftertaste-A was significantly positively correlated with coptisine, epiberberine, berberine, and palmatine (*P <*0.01), suggesting that astringency and aftertaste-A could be used as quality evaluation indexes of CR. Moreover, saltiness did not correlate with alkaloids, which is consistent with the saltiness result of the electronic tongue in CR.

**Table 4:** The correlation analysis of tastes and alkaloids for *Coptis* rhizome.

Variable	Sourness	Bitterness	Astringency	Aftertaste-B	Aftertaste-A	Umami	Richness	Saltiness
Magnoflorine	0.536^*^	-0.360	0.583^*^	0.321	0.589^*^	-0.297	-0.259	0.148
Tetrahydricheilanthifolinium	0.309	0.128	0.591^**^	0.22	0.745^**^	-0.282	-0.581^*^	-0.358
Coptisine	0.341	0.132	0.525^*^	0.243	0.738^**^	-0.289	-0.482^*^	-0.295
Epiberberine	0.362	0.107	0.628^**^	0.181	0.808^**^	-0.334	-0.563^*^	-0.337
Columbamine	0.342	0.136	0.401	0.349	0.798^**^	-0.244	-0.156	-0.07
Jatrorrhizine	0.038	-0.486^*^	-0.087	-0.203	-0.302	0.074	-0.152	0.071
Berberrubine	0.479^*^	-0.081	0.453	0.493^*^	0.728^**^	-0.314	-0.364	-0.093
Berberine	0.602^**^	-0.094	0.612^**^	0.464	0.841^**^	-0.441	-0.189	0.095
Palmatine	0.346	0.093	0.569^*^	0.254	0.783^**^	-0.285	-0.514^*^	-0.285
Berlambine	0.137	-0.155	-0.347	0.333	0.153	0.133	0.238	0.192

* means *P <*0.05, ** means *P <*0.01, and “-” indicates a negative correlation.

## 4 Discussion

The technology of taste evaluation based on the material has highlighted the characteristics of traditional Chinese medicine modernization. This study found that the electronic tongue technique can be used as a new method to evaluate the quality of CR, consistent with previous research opinions (Ran et al., 2024). The electronic tongue can realize the objectification and visualization of taste indicators, which has the advantage of faster and more convenient operation than the LC-MS method. In addition to CR, there are a variety of Chinese medicine applications of electronic tongue research reports, such as *Schisandrae Chinensis* fruit (Wang L. et al., 2024), *Rheum* root and rhizome (Liu et al., 2024), and *Bletilla striata* rhizome (Li H. et al., 2024), etc.

CR is a multi-source herbal medicine, suggesting that treating them differently in quality evaluation is necessary. However, the clinical and pharmaceutical enterprises’ use of CR decoction pieces does not distinguish from which species, and the real-world view is that their effects are the same. According to the results of electronic tongue and mass spectrometry detection, there are differences among the four kinds of CR. Uniformly, OPLS-DA and HCA showed that taste values and alkaloid contents could distinguish CR from different plant sources. These findings are consistent with the quality requirements of CR in the Chinese Pharmacopoeia; that is, the alkaloid types and content requirements corresponding to different species of CR are different. Further correlation analysis showed a correlation between tastes and alkaloid contents in CR. In particular, the chemical markers, such as coptisine, epiberberine, berberine, and palmatine, for the quality evaluation of CR are associated with CR’ tastes (aftertaste-A, astringency, aftertaste-B) a positive correlation or significant positive correlation, which addressed the lack of correlation study between the CR’s specific alkaloid types and the electronic tongue characterization of “bitter taste” in the previous study (Zhang et al., 2017).

In the theory of traditional Chinese medicine, the “five tastes” of Chinese medicines represent their authentic taste and, on the other hand, represent some of the effects. CR is a bitter Chinese medicine. It has the traditional functions of clearing heat, draining fire, and drying dampness. It shows the modern pharmacological effects, such as anti-inflammation and anti-diarrhea (Li C. et al., 2024; Hu et al., 2024). The traditional view is that the more bitter the taste of CR, the better its quality. This study’s results also confirm that the higher the aftertaste-B value of CR, the higher the content of berberrubine in CR. It has been reported that the total alkaloids of CR inhibited the PI3K-AKT pathway and reduced inflammation by activating PPARγ (Li J. et al., 2024). Berberine alleviates intestinal inflammation in mice by improving endoplasmic reticulum stress (Yarmohammadi et al., 2022) and immunomodulatory effects (Li et al., 2020). It is speculated that higher aftertaste-B and higher content of specific alkaloids have better anti-inflammatory effects. In this study, we analyzed the correlation between the tastes of CR and its alkaloids, which are the representatives of the pharmacodynamic activity of CR. Therefore, through the axis of taste-alkaloid-pharmacological activity of CR, the author proposed a preliminary and predictive conclusion between the taste and pharmacological effect of CR. Of course, more experiments are needed to verify it at the next step.

## 5 Conclusion

In this study, the electronic tongue technique was successfully used to achieve the objective evaluation of CR taste, and it can also be used to distinguish CR from different plant sources. In combination with LC-MS results, the correlation between tastes and alkaloids was established, which provided a new idea for the quality evaluation of CR. However, due to resource protection, the number of CD, CT, and CJ samples is small, which leads to limitations on the research results.

## Data Availability

The raw data supporting the conclusions of this article will be made available by the authors, without undue reservation.
